# NLRP3 neuroinflammatory factors may be involved in atopic dermatitis mental disorders: an animal study

**DOI:** 10.3389/fphar.2022.966279

**Published:** 2022-10-04

**Authors:** Huimin Yuan, Yan Sun, Shujing Zhang, Jing Feng, Zijiao Tian, Jingang Liu, Hang Wang, Yushan Gao, Yang Tang, Fengjie Zheng

**Affiliations:** School of Traditional Chinese Medicine, Beijing University of Chinese Medicine, Beijing, China

**Keywords:** atopic dermatitis, mental disorders, neuroinflammation, NLRP3, animal model

## Abstract

**Background:** Numerous clinical studies have shown that atopic dermatitis (AD) is often associated with mental disorders. This could contribute to the overall burden of atopic dermatitis. However, the underlying mechanism of mental health symptoms in AD has not been fully elucidated.

**Methods:** An AD mouse was induced by 2,4-dinitrofluorobenzene (DNFB), which was repeatedly applied to the back skin of the BALB/C mice to establish an atopic dermatitis mental disorder model. The role of neuroinflammation in the pathogenesis of atopic dermatitis mental disorders was then explored.

**Results:** After the stimulation of DNFB for 35 days, the skin lesions, the HE staining of skin lesions, and the behavioral experiments (including elevated plus maze assay and tail suspension test) suggested that the AD mental disorder mouse model was successfully replicated. The expression of neuroinflammatory factors in the hippocampus was then investigated through Western blotting. The results showed a significant increase in the protein expression of NLRP3, caspase-1, and IL-1β.

**Conclusion:** Mental disorders in AD might be related to the neuroinflammatory response in the hippocampus. An alternative yet essential approach to promoting AD recovery could be through reducing neuroinflammation and improving mental disorders.

## Introduction

Atopic dermatitis (AD), also known as ectopic dermatitis, is a chronic, recurrent, and inflammatory skin disease characterized by severe itching and eczema-like lesions. This disorder affects people of all ages and ethnicities and has a substantial psychosocial impact on patients, which is the leading cause of the global non-health burden of skin disease (Weidinge and Novak, 2016). A large number of studies have found that intense itching and recurrent skin lesions will lead to anxiety- and depression-like mental disorders in patients, seriously affecting the quality of life of patients (Schonmann et al., 2020; Silverberg, 2019). In addition, mental disorders may also result in scratching behaviors that destroy the skin barrier, hence contributing to the vicious cycle involved in the progression of AD (Akdis et al., 2006; Sanders and Akiyama, 2018; Silverberg et al., 2019). Proper disease management is needed to improve emotional disorders in AD (Silverberg, 2019). While various AD animal models have been used successfully, few of them have reported on emotional characteristics which suggests that the pathogenesis of AD with a mental disorder has not been fully uncovered. Hence, basic studies elucidating the mechanisms contributing to AD with a mental disorder will allow for creating more personalized and efficient treatment approaches.

Recent studies show that the nod-like receptor protein 3 (NLRP3) inflammasome is associated with the pathogenesis of depression-like and anxiety-like behaviors (Kaufmann et al., 2017; Su et al., 2017; Talukdar et al., 2021). The NLRP3 inflammasome is a multimeric protein complex mainly present in macrophages and hippocampal microglia, including procaspase-1 precursor and apoptosis-associated speck-like protein adaptors, which is the main mediator promoting the processing and secretion of IL-1β and plays a critical cytokine role in neuroinflammation response (Talukdar et al., 2021; Swanson et al., 2019; Schroder and Tschopp, 2010). Meanwhile, many experiments also proved that NLRP3 is a key target of mental disorders. It is reported that depression can be improved by inhibiting NLRP3 (Zhang et al., 2019; Chen et al., 2021; Li et al., 2021). However, to our knowledge, the NLRP3 inflammasome has not been reported to be involved in the pathological process of anxiety and depression in AD.

Currently, the main purposes of AD treatment are to alleviate or eliminate clinical symptoms, eliminate triggers and/or aggravating factors, decrease and prevent a recurrence, reduce or alleviate comorbidities, and improve the quality of life of patients (Chen, 2020). A proper protocol for the treatment and management of AD can significantly improve and reduce its symptoms, allowing patients to enjoy life. Presently, there are not many clinical studies on psychological disorders like depression and anxiety associated with AD. Of the few published, it has been found that the use of dupilumab and abrocitinib can not only alleviate the symptoms of moderate-to-severe AD but also help reduce anxiety and depression, improving patients’ quality of life (Cork et al., 2020; Silverberg et al., 2021). In other studies, antidepressants such as mirtazapine and paroxetine have been found to help relieve persistent itching and improve sleep quality, albeit with some side effects (Wollenberg et al., 2018). Despite results showing that concurrent treatment of skin conditions and mental disorders can allow for major improvements in patients’ quality of life, studies in this field are still limited.

The NLRP3 inflammasome has been widely recognized as an important target for mental disorders such as anxiety and depression. Studies have found that rapamycin can decrease anxiety and depression in pentylenetetrazole-kindled mice by regulating the NLRP3 pathway (Aghaie et al., 2021). Isoliquiritigenin is a chemical compound found in Gan Cao (*Glycyrrhiza uralensis*). It has been reported that isoliquiritigenin can suppress NLRP3-mediated pyrophosphorylation to improve depression (Li et al., 2021). These herbs/drugs can be used as co-therapy for AD with anxiety and depression, improving the effectiveness of AD treatment. In the previous experiments, we discovered that DNFB-induced AD mice exhibited anxiety- and depression-like behaviors (unpublished). In this study, we will dynamically observe the emotional characteristics of the AD mouse model at different times and the expression of neuroinflammatory factors related to NLRP3, in order to reveal the potential mechanism of AD with mood disorders. Based on the NLRP3 inflammasome, not only can we provide more potential therapeutic targets such as co-therapy for AD with anxiety and depression but also provide a reference for exploring new drugs for AD therapy.

## Materials and methods

### Approvals

The use and disposal of the experimental animals in this study met the requirements of animal welfare. Experiments were carried out in the animal laboratory of the Beijing University of Chinese Medicine after the approval of the Animal Ethics Committee of the Beijing University of Chinese Medicine, China.

### Animals

Six-week-old specific-pathogen-free BALB/c male mice (*n* = 48) with body weight (20 ± 2) g were purchased from Beijing Vital River Laboratory Animal Technology Co., Ltd. [license number: SYXK (Beijing) 2016-0006]. They were housed in IVCs in the animal laboratory of the Beijing University of Chinese Medicine, at an ambient temperature of (25 ± 2)°C and humidity of 40%–60%, and they had free access to food and water daily.

After 7 days of adaptive feeding, the mice were randomly divided into two groups: *n* (AD) = 24 and *n* (CONT) = 24. A total of six mice from each group were sacrificed on days 11, 25, 39, and 46 during the experiment.

### Reagents

2,4-Dinitrofluorobenzene (DNFB) was purchased from Shanghai Macklin Biochemical (No. M24218031; Shanghai, China). The NLRP3 antibody was purchased from Abcam (No. Ab214185; Shanghai, China), the anti-cleaved caspase-1 antibody was purchased from CST (No. 89332S; Shanghai, China), the IL-1β monoclonal antibody was purchased from Proteintech (No. 66737-1-Ig; Hubei, China), and the goat anti-rabbit IgG/HRP and Rb a beta-actin antibodies were purchased from Beijing Biosynthesis Biotechnology (No. BJ08079044, No. AH11286487; Beijing, China). The hematoxylin staining solution and eosin staining solution were purchased from Beijing Zhong Shan-Golden Bridge Biological Technology (No. 20042002 and No. 20060901; Beijing, China). The bicinchoninic acid protein quantification kit was purchased from Beijing Pulilai Gene Technology (No. 2019N1AP1511; Beijing, China), and the electrochemiluminescence (ECL) light-emitting solution was obtained from Millipore (No. 20136B2; Billerica, MA, Unites States).

### Instruments

An animal behavior video acquisition system was supplied by SONY (China) Co., Ltd. (SSC-G213, Guangzhou, China). An animal video behavior analysis system and an elevated plus-maze facility were supplied by Beijing Zhongshi Dichuang Science and Technology Development Co., Ltd. (smart 3.0, 1056306, 1056012; Beijing, China). An automatically closed tissue dehydrator, a paraffin-embedding machine, a tissue-sectioning cold table, an automatic slicer, a spreader, and a baking machine were supplied by Leica (Brunswick, Germany). A forward-inverted integrated fluorescence microscope was provided by Echo (San Diego, CA, United States), a frozen high-throughput tissue grinder was provided by Ningbo Scientz Biotechnology (Ningbo, China), an electrophoretic apparatus and a T100 thermal cycler were supplied by Bio-Rad (Hercules, CA, United States), and an ultra-sensitive multi-functional imager was provided by Azure Biosystems (Dublin, CA, United States).

### Establishment of the atopic dermatitis model

**FIGURE 1:**
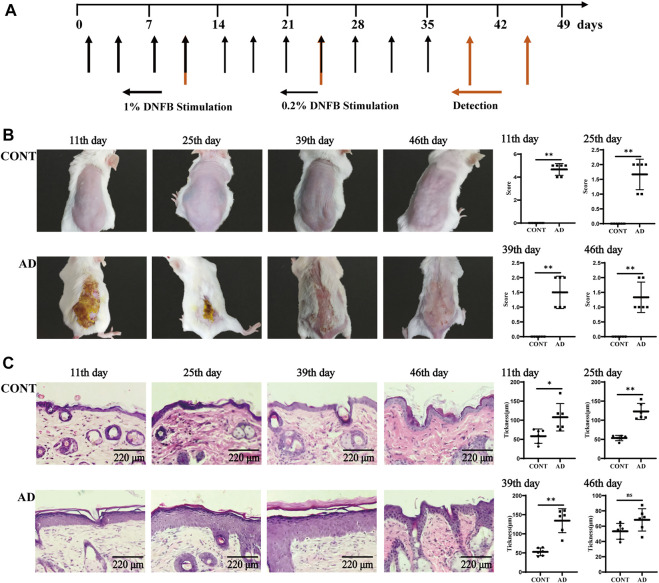
Experimental flow chart and skin observation. **(A)** Time flow chart of DNFB stimulation and detection. The morphology and functions of the skin and hippocampus and behavioral experiments, such as OFT and TST, were operated on the 11th, 25th, 39th, and 46th day after primary stimulation. **(B)** Back skin performance and the skin lesion scores in mice at different times (because of the long fur hair, the shaving operation was carried out again before the 39th and 46th day, and the specific shaving operation was the same as before). **(C)** Skin histology and epidermal thickness in mice at different times. All data are presented as mean ± SD; *n* = 6 mice/group. *p < 0.05, **p < 0.01 *vs.* the CONT group. CONT, normal mice; AD, DNFB-treated mice.

The mouse model of atopic dermatitis was prepared by repeated stimulation of DNFB hapten (Kim et al., 2013; Yoou et al., 2018). Specific operations were as follows: one day before DNFB sensitization, an area measuring about 2.0 cm × 3.0 cm of the back hair of mice was removed with a push-shear and hair removal cream. During stimulation, 200 ul of 1% DNFB solution was dripped on a 2 cm × 2 cm gauze and evenly smeared on the back skin of the mouse twice a week (that is, 1st, 4th, 8th, and 11th day of the modeling). After 2 weeks, 0.2% DNFB solution sensitization stimulus was used, and the aforementioned operation was repeated twice a week for 3 weeks (that is, 15th, 18th, 21st, 25th, 28th, 32nd, and 35th day). The DNFB hapten sensitization stimulation lasted for 5 weeks (Figure 1A). Note that the time and frequency of stimulation in the blank group were the same as that of the model group.

### Tissue preparation

On the 11th, 25th, 39th, and 46th day, six mice in each group were subjected to an elevated plus maze assay (EPM) test and tail suspension test (TST). We then anesthetized them with 2% pentobarbital sodium, removed their blood, and separated serum and skin tissue for detection and histological observation. The tissue of the hippocampus was removed for screening under sterile conditions.

### Observation of skin lesions

Before each skin sensitization, the skin changes were visually observed and photographed and recorded. They were then graded in four aspects: hemorrhage/erythema, scarring/dryness, edema, and epidermal exfoliation/erosion/lichenification (Tian et al., 2019).

### Skin histopathology and epidermal thickness determination

The formalin-fixed skin tissue samples were embedded in paraffin, cut into 6-μm-thick sections, and stained with HE for histological examination. At each time point, six different fields of view were selected from the HE-stained sections of the mice in each group and six points were randomly selected in each field of view. The thickness of the skin lesions on the back of the mice was measured sequentially using ImageJ software (version 1.52a; National Institutes of Health, Bethesda, MD, United States), and the results were averaged.

### Elevated plus maze assay

Experimental device: the wooden cross maze includes three parts: two opposite open-arm areas (30 cm × 6 cm), two relative closed-arm areas with vertical walls (30 cm × 6 cm × 15 cm), and a central area (6 cm × 6 cm). The cross maze is 75 cm high from the ground, and it is illuminated by double cold lights 40 cm high from the central area.

Operations in the experiment: the mice were placed in the open space for 5 min before the test. The operator placed the mice in the EPM (central zone, head to open-arm area) and recorded the behavioral changes of the animals for 5 min with the camera system, including the number of times the mice entered the open-arm entry, closed-arm entry, and central zone as well as the dwelling time they spent in the open entry, closed-arm entry, and central zone. In this case, experimental parameters were recorded when all four paws of the mice entered from one arm area into another. At the end of each animal’s experiment, the feces were removed, and the bottom of the box was sprayed with 75% ethanol and dried using a clean gauze to avoid influencing the experimental results of the following mice with the residual odor of the previous animals. The tests were conducted from 9:00 a.m. to 12:00 a.m. At the end of the experiments, the data were extracted and recorded by personnel unfamiliar with the experimental design. The percentage of open-arm time (Otime% < 20%) and percentage of open-arm entries (Oentries% < 25%) were used as indices of anxiety-like behavior (Luo et al., 2019). The number of times they entered the open-arm entries and the length of the dwelling time were negatively correlated with the anxiety of mice. The fewer the number of times they entered the open-arm entries and the shorter the dwelling time they spent, the more anxious the mice were.

The EPM was included in the experimental metrics:

1) Otime%: the percentage of the open-arm dwelling time [open-arm area dwelling time/(open-arm area dwelling time + closed-arm area dwelling time) × 100%].
2) Oentries%: the percentage of open-arm entries [open-arm area entries/(open-arm area entries + closed-arm area entries) × 100%].

### Tail suspension test

The tail suspension test was carried out 30 min after the EPM test. In the experiment, every mouse was hung for 5 min with its tail fixed to a tape about 2 cm. At the same time, an experimenter recorded the immobility time of the mouse, which reflected its despair and depression to some degree.

### Western blotting

Hippocampus tissues were lysed with an appropriate amount of radioimmunoprecipitation lysate containing phenylmethanesulfonyl fluoride and grinding beads in a tissue grinder. The supernatant was then extracted using a centrifuge, and the denaturation was carried out through bicinchoninic acid protein quantification. Proteins were separated by SDS-PAGE gel electrophoresis and blocked by a 5% skim milk powder after constant pressure electrotransfer to the polyvinylidene difluoride membrane. The membrane was incubated overnight with primary antibodies, then incubated with secondary antibodies after washing three times with TBST, and eventually developed with ECL chemiluminescence after washing with TSBT three times. ImageJ (version 1.52a; National Institutes of Health, Bethesda, MD, United States) was then used to analyze grayscale values, and it calculated the relative expression with target protein grayscale values/β-actin grayscale values.

### Statistical analysis

All data were presented as means ± SD. An unpaired two-tailed *t*-test was used to compare the means of the two groups. Pearson correlation and linear regression analysis were used to assess the relationships between the behavioral results and gene levels of neuroinflammatory-related factors. All statistical analyses were performed in Prism version 9.0 (GraphPad, California, SD, United States). The value of *p* < 0.05 was considered to be statistically significant.

## Results

### Skin lesion and histopathology

To evaluate the AD model, we observed and analyzed the mouse skin and histopathological states at different times (Figure 1B). The performances of sensitized skin injury on the back of the mice in the model group on the 11th, 25th, 39th, and 46th day of modeling and at different time points during replication showed their skin lesions with a pathological process similar to the clinical one. The acute phase (1–11 days) consisted of erythema and hemorrhagic spots → edema and ulceration of the skin → new blood scabs → hardening of the scabs; the acute progressive phase (12–25 days) showed the coexistence of blood scab shedding and new scarring. The subacute phase (26–39 days) showed scattered erythema and hemorrhagic spots, scarring, and scaling or mossy dryness, etc., and the chronic phase (after 39 days) was mainly characterized by dry skin. Compared to the normal group, skin lesion scores were significantly high in the AD model group mice at all time points throughout the observation cycle (each *p* = 0.002).

As for histological changes (Figure 1C), the skin of normal mice had regular structure, normal cell morphology, and clear skin hierarchy. The skin of AD mice, however, showed the following sequence: thickening of the epidermis, mainly thickening of the echinocyte layer → thickening of the epidermis and echinocyte layer → thickening of the epidermis and significant thickening of the echinocyte layer → less thickening of the epidermis and less proliferation of the echinocyte layer. Compared to the normal group, skin epidermis thickness was significantly high in the AD model group mice on the 11th (*p* = 0.013), 25th (*p* < 0.000), and 39th day (*p* = 0.000).

### Elevated plus maze assay, tail suspension test, and weight

**FIGURE 2:**
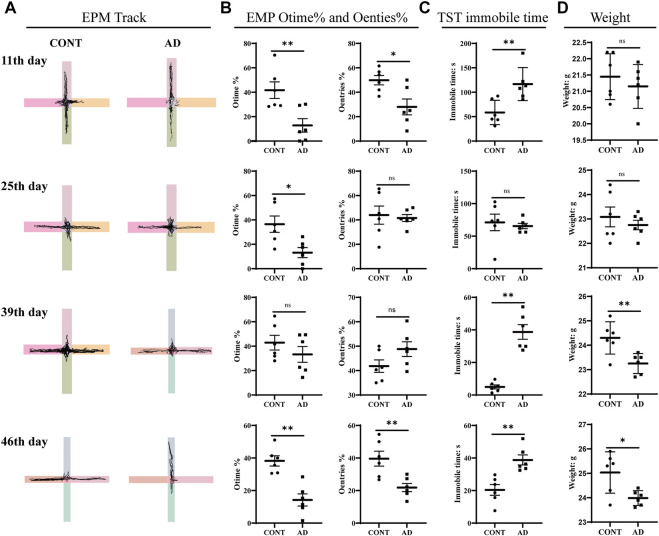
EMP, TST, and weights. **(A)** EPM track, the horizontal axis is the open arm and the vertical axis is the closed arm. **(B)** Results of EMP Otime% and Oentries%. **(C)** TST immobile time. **(D)** Weights: all data are presented as mean ± SD; *n* = 6 mice/group. *p < 0.05, **p < 0.01 *vs*. the CONT group. CONT, normal mice; AD, DNFB-treated mice.

To determine the emotional disorder state of mice with AD replicated by DNFB, we conducted behavioral tests such as EPM and TST as well as weighing at different times (Figure 2). We first tested mice for anxiety-like behavior using the EPM. The DNFB-induced AD mice spent significantly less time in the open-arm area and entered into it less times than the controls on the 11th day (*P*_Otime%_ = 0.008 and *P*_Oentries%_ = 0.016) and 46th day (*P*_Otime%_ = 0.001 and *P*_Oentries%_ = 0.007). We then assessed depression-like behaviors using the TST, a measure of behavioral despair, and evaluated the depressive condition through the weight of the mice. The DNFB-treated mice showed a pronounced increase in behavioral despair when compared with the normal mice, as seen in the significant increase in time spent immobile in the TST on the 11th (*p* = 0.007), 39th (*p* < 0.000), and 46th day (*p* = 0.002). The normal group increased steadily throughout the experiment, while the mice in the model group were significantly lighter on the 39th (*p* = 0.008) and 46th day (*p* = 0.017), showing statistically significant differences. These could indicate that atopic dermatitis model mice induced by DNFB exhibited significant behavioral manifestations of anxiety-like and depression-like mental disorders on days 11 and 46 of the experiment.

### Protein expression of nod-like receptor protein 3, caspase-1, and IL-1β

**FIGURE 3:**
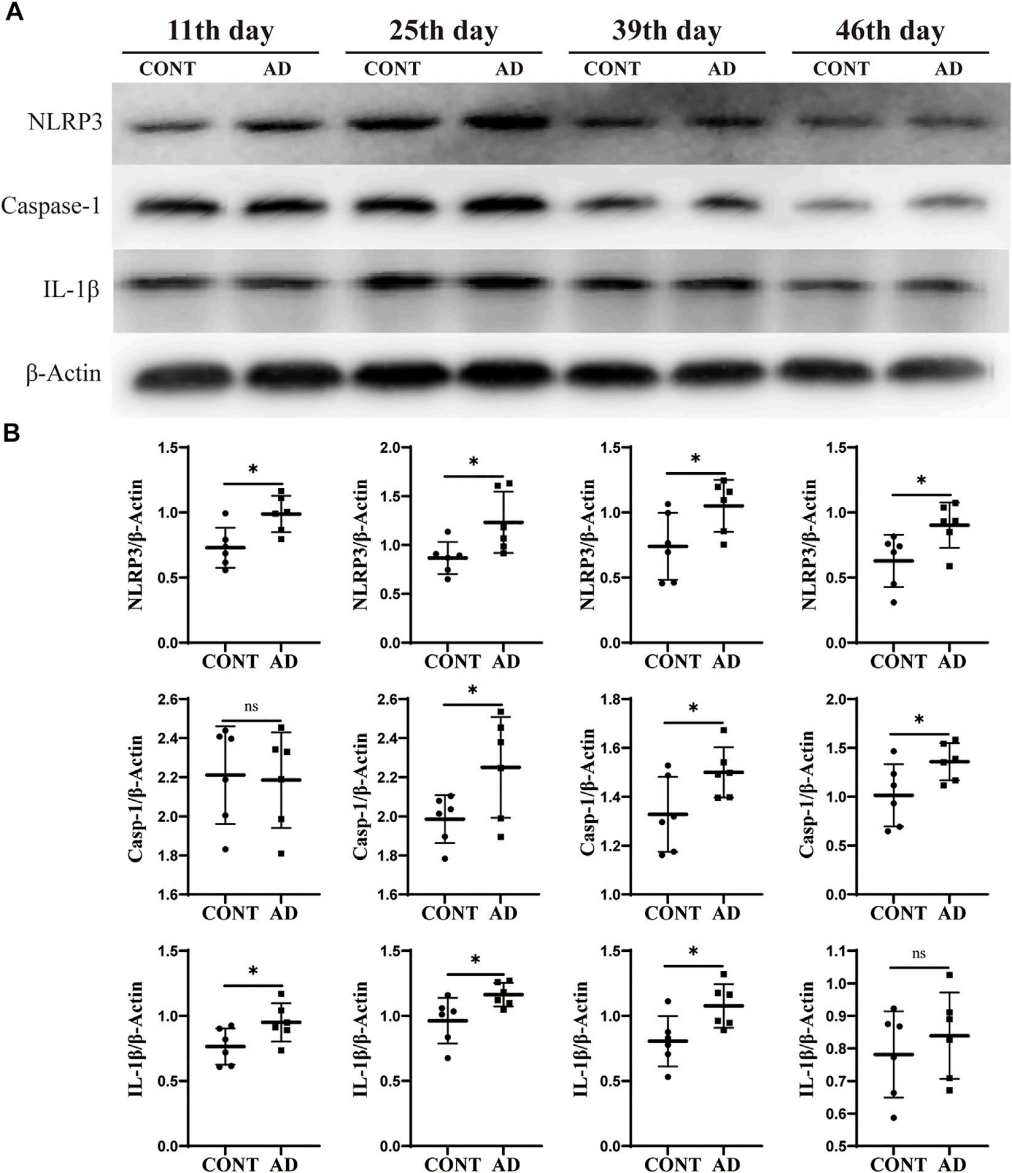
Protein expression of NLRP3, caspase-1, and IL-1β. **(A)** Western blotting of NLRP3, caspase-1, and IL-1β expression in mice hippocampus at different time points. **(B)** Comparison of relative gray values of NLRP3, caspase-1, and IL-1β in mice hippocampus at different time points. Data are expressed as mean ± SD; *n* = 6 mice/group. *p < 0.05 *vs.* CONT group. CONT, normal mice; AD, DNFB-treated mice.

To determine the related neuroinflammation in the hippocampus of DNFB mice, the protein expression screening of NLRP3, caspase-1, and IL-1β was conducted at four different time points. Also, it was observed that NLRP3, caspase-1, and IL-1β expression was increased in varying degrees in the model group compared to those in the control group on the 11th, 25th, 39th, and 46th day (Figure 3). The AD group showed statistically significant differences in NLRP3 on the 11th day (*p* = 0.012), 25th day (*p* = 0.030), 39th day (*p* = 0.042), and 46th day (*p* = 0.030). Statistically significant differences in caspase-1 were also observed on the 25th day (*p* = 0.047), 39th day (*p* = 0.046), and 46th day (*p* = 0.047). Meanwhile, in terms of IL-1β, the AD group showed statistically significant changes on the 11th day (*p* = 0.049), 25th day (*p* = 0.032), and 39th day (*p* = 0.027).

### Correlation between behavioral results and neuroinflammation

**FIGURE 4:**
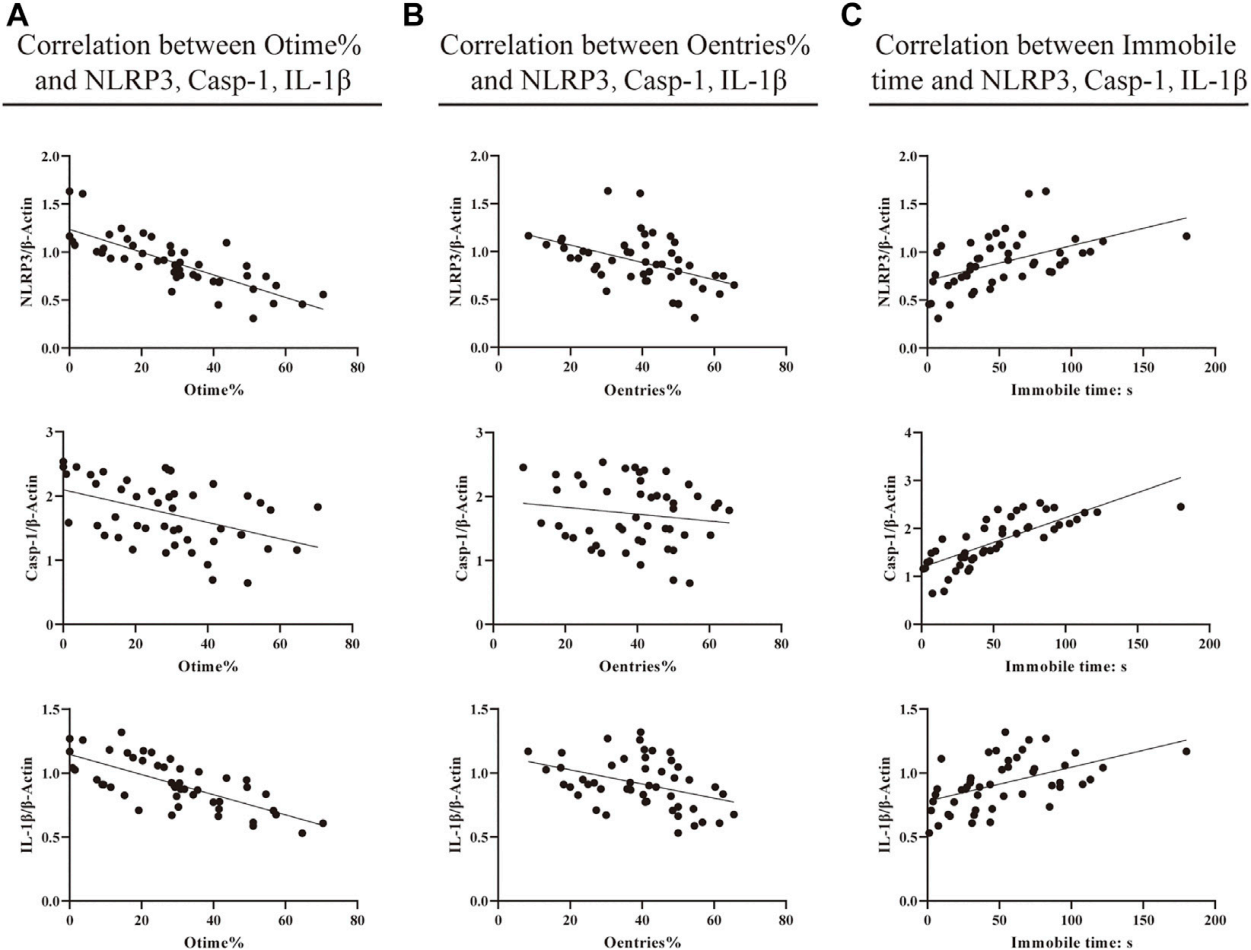
Correlation between behavioral results and neuroinflammation. **(A)** Correlation between EMP Otime% and protein expression of NLRP3, caspase-1, and IL-1β. **(B)** Correlation between EMP Oentries% and protein expression of NLRP3, caspase-1, and IL-1β. **(C)** Correlation between TST immobile time (s) and protein expression of NLRP3, caspase-1, and IL-1β (Pearson correlation and linear regression analyses were performed to test potential associations of the EPM and TST with protein expression levels of NLRP3, caspase-1, and IL-1β in the brain hippocampus of mice. Values of 0.3 ≤ |r| ≤ 0.5 were considered statistical differences, and 0.5 ≤ |r| ≤ 0.8 were considered significant differences).

To observe the relationship between the indicators, we performed a correlation analysis (Figure 4). The results indicated that there was a significant correlation between the EMP [the percentage of time the mice spent in open arms (Otime%) and the percentage of the times they entered open-arm entries (Oentries%)], TST (the immobile time), and neuroinflammation (NLRP3, caspase-1, and IL-1β protein expression, respectively). The corresponding findings were as follows: there were negative correlations between NLRP3 protein expression and Otime% and Oentries% (*r* = −0.783, *p* < 0.000; *r* = −0.464, *p* = 0.001), between caspase-1 protein expression and Otime% (*r* = −0.451 and *p* = 0.001), and between IL-1β protein expression and Otime% and Oentries% (*r* = −0.713, *p* < 0.000; *r* = −0.390, *p* = 0.006). Also, there were positive correlations between NLRP3, caspase-1, and IL-1β protein expression and immobile time (*r* = 0.505, *p* = 0.000; *r* = 0.777, *p* < 0.000; and *r* = 0.506, *p* = 0.000). This suggests that the increase in the protein expression of NLRP3, caspase-1, and IL-1β may have participated in anxiety-like and depression-like behavioral phenotypes in mice with atopic dermatitis.

## Discussion

In recent years, there has been increasing evidence showing the bidirectional relations between psychological factors and AD (Fabrazzo et al., 2021; Huet et al., 2022). However, there are very few animal experimental studies on this subject, leaving two key points unclear, namely, the characteristics of negative mental states and their underlying mechanism in AD mice. In this study, we investigated the characteristics of emotion using an AD mouse model at four different time points, with a particular focus on the expression of neuroinflammation linked with NLRP3.

First, the mice in the model group were induced by DNFB. No other methods of stimulating mental disorders, such as LPS treatment or chronic unpredictable mild stress (Wang et al., 2020; Willner, 2016), which were used by Li et al. (2016), were implemented. We found that the mice showed clinical characteristics of AD from the 11th day to the 46th day after primary stimulation. To better study the characteristics of emotional disorders, we observed the behavioral changes of AD at four different points.

The EPM and TST were used as classic behavioral test methods to study and evaluate the anxiety and depression behaviors of animals (Hao et al., 2019). In this study, we found that AD mice showed more significant anxiety- or depression-like behavioral performances on the 11th, 25th, 39th, and 46th day. However, it should be acknowledged that the behavioral results of the mice on the 25th day and 39th day were not stable, whose results were not completely consistent with the EPM and TST. Some studies have shown that chronic stressful events are documented as a vital cause of both depression and anxiety disorders, and stress from external chronic itching, pain, and other perceptual stimuli can stimulate stressful mental disorders and behavioral manifestations in experimental animals (Tang et al., 2019). According to the AD model preparation process, we found that on the 11th day of the experiment when AD mice were sensitized by high-concentration DNFB for the last time, their stress behaviors were prominent, with severe skin lesions and a significant increase in the number of scratching (Li et al., 2021). Therefore, although the EMP results at this time suggest that AD mice may have anxiety-like behavioral manifestations, it is also possible that AD mice are stimulated by DNFB to produce stressful behaviors. When the sensitized state stimulation was maintained at a low concentration of 0.2% DNFB, AD mice may show adaptation to DNFB stimulation, resulting in no significant anxiety-like behavior on the 25th and 39th day of the EMP test. In particular, the Oentries% of AD mice was higher than that of the control group on the 39th day of modeling, although there was no statistical difference in the comparison of Otime% and Oentries%. This may be related to the adaptation of AD mice to DNFB stimulation or insufficient statistics. Interestingly, on the 46th day of the experiment without DNFB stimulation, the EMP results showed that AD mice had the possibility of anxiety-like behavior. Due to the animals being continuously stimulated by DNFB from the first day to the 35th day, we speculate that it may happen in the adaptive stage after starting or stopping stimulation on the 25th day and 39th day. If so, it may be a good state to study the AD mood disorder after the 46th day.

Studies (da et al., 2021; Yeom et al., 2020) had shown that AD mice could exhibit anxiety-like mental disorders related to the mechanism of neuroinflammation in hippocampi. However, not much research focused on the NLRP3 inflammasome, which plays a critical role in neuroinflammation response to develop mental disorders (Talukdar et al., 2021; Swanson et al., 2019; Schroder, 2010). In this study, we found that the expression of NLRP3, caspase-1, and IL-1β in the hippocampus of the AD mice was significantly higher than those in the mice of the normal group on the 11th, 25th, and 39th day. This suggests that they may be important factors in the regulation of the negative emotions of AD. Surprisingly, on the 46th day, although there was no significant difference in the expression of IL-1β between the AD group and the normal group, NLRP3 and caspase-1 continued to show an increasing trend. In addition, we also found that the expression of caspase-1 was associated with the week’s age in mice.

The correlations between these inflammatory factors and behaviors were analyzed. Results showed that the protein expression of NLRP3 was negatively correlated with Otime% and Oentries% and positively correlated with the resting time of the tail suspension experiment. At the same time, the protein expression of caspase-1 was negatively correlated with the percentage of residence time in the open-arm area and positively correlated with the immobile time in the TST. The protein expression of IL-1β was negatively correlated with Otime% and Oentries% and positively correlated with the immobile time in the TST. This suggests that NLRP3, caspase-1, and IL-1β may be involved in the anxiety-like and depression-like behavioral phenotypes of AD mice.

In fact, AD associated with psychological stress disorders such as anxiety and depression have aroused widespread concern in clinics. We believe that the mechanism of mental disorders in atopic dermatitis is complex. On the one hand, it activates a specific mechanism due to itching. On the other hand, mental disorders aggravate itching, forming a vicious circle. Hence, if mental disorders have developed, concurrent treatment of both skin and psychological disorders will allow for the most favorable prognosis. We have noticed the reports that dupilumab and abrocitinib can also reduce anxiety and depression and improve the quality of life in addition to improving the symptoms of moderate-to-severe AD (Cork et al., 2020; Silverberg et al., 2021). As a critical component in the treatment of AD, this is achieved mainly through improving skin conditions, relieving itch, and thereby, reducing anxiety and depression. However, if deeply set mechanisms of mental stress have already been formed, AD may and will relapse even without triggers. Hence, some scholars have sought to improve the prognosis of AD through treatment of anxiety and depression; for instance, the usage of antidepressants such as mirtazapine and paroxetine has clinically been found to help relieve persistent itching while improving psychological symptoms, albeit with some side effects (Wollenberg et al., 2018). It is rather unfortunate that studies on this mechanism have been scarce, especially those conducted through a dynamic approach. The discovery of the classic target NLRP3 in our study will serve a major role in upgrading AD treatment plans in the future. Many drugs have already been found to target NLRP3. Studies have shown that rapamycin can decrease anxiety and depression in pentylenetetrazole-kindled mice by regulating the NLRP3 pathway (Aghaie et al., 2021). Isoliquiritin, a chemical compound found in Gan Cao (*Glycyrrhiza uralensis*) has been found to suppress NLRP3-mediated pyrophosphorylation, reducing depression (Li et al., 2021). It has also been reported that traditional Chinese medicine monomers such as resveratrol and albiflorin can help to control hippocampal inflammation through the suppression of the NLRP3 inflammasome, reducing anxiety and depression (Liu et al., 2019; Liu et al., 2021). Furthermore, Shugan Hewei Decoction containing Chai Hu (*Bupleuri radix*), Huang Lian (*Coptis chinensis*) and Gan Cao (*Glycyrrhiza uralensis*) has been reported to help manage anxiety and depression in chronic stress model rats by suppressing the NLRP3 inflammasome and cecal microbiota (Yue et al., 2021). The aforementioned medicine/drugs/chemical compounds may all be potential supplementary medicines in the treatment of psychological stress and in enhancing the therapeutic effect of AD patients. In addition, since it has been proven that NLRP3 inflammasomes participate in the pathological pathway of AD skin disorders, they can serve as a reference to screen for drugs that can target both skin inflammations and psychological stress. Therefore, we believe that the consistent and active observation of the NLRP3 inflammasome and its inflammatory factors will provide inspiration for the screening of AD drug intervention in the future.

Our study also had some limitations. The results of behavioral experiments were inconsistent at different stages. We suspect that it may be due to DNFB stimulation, but further exploration needs to be conducted to identify the specific reasons. Furthermore, the duration of anxiety- or depression-like mental disorder in AD mice remains unclear, especially after the 46th day. A more in-depth investigation could be conducted in this area. Notably, we found that NLRP3-related factors were expressed at different stages, suggesting that they may be involved in the mental disorder of AD. The expression of NLRP3-related factors was not completely consistent with the behavioral tendency. On the 46th day, the behavioral performance was stable. The expression of NLRP3 and caspase-1 showed a consistent upward trend, while no significant difference was observed in the expression of IL-1β, which suggests that other mechanisms were involved or had inhibited its expression. It is really a matter of concern that the skin is not only a target organ of multiple hormones but also an endocrine organ for the secretion of multiple hormones, with equivalent functions of the hypothalamic−pituitary−adrenal (HPA) axis (Slominski and Mihm, 1996; Slominski and Wortsman, 2000; Lin et al., 2017). The skin neuro-endocrine-immune system interacts bidirectionally with the central nervous system, endocrine system, and immune system to maintain the body’s homeostasis (Slominski and Wortsman, 2000; Jin et al., 2022). Upon perception of external or endogenous stimuli, the HPA axis of the skin and endocrine system will be activated (Alexopoulos and Chrousos, 2016; Slominski et al., 2007), which is followed by the release of a corticotropin-releasing hormone (CRH) of the hypothalamus and the skin (Skobowiat and Slominski, 2015). CRH then upregulates the expression of toll-like receptors (TLRs) and acts on the skin nerve fiber receptors, which amplifies the skin nerve–immune system interaction and eventually leads to the occurrence of inflammation (Slominski et al., 2013; Liu et al., 2012). Indeed, our study only covered the correlation between nerve and skin, while the details in between remained unfocused. Whether the NLRP3 inflammasome affects the inflammatory response of the skin through the aforementioned pathway will require further research in the future. The small animal sample size may also be a factor contributing to the incomplete consistency of related trends.

## Conclusion

To sum up, AD mice induced by DNFB appeared with mental disorders, as early as the 11th day. On the 46th day, they may be in a stable state. NLRP3-related inflammatory factors may be a potential target of emotional disorders in AD.

## Data Availability

The original contributions presented in the study are included in the article/Supplementary Material; further inquiries can be directed to the corresponding authors.

## Abbreviations

AD, atopic dermatitis; DNFB, 2,4-dinitrofluorobenzene; EMP, elevated plus maze assay; NLRP3, nod-like receptor protein 3; Oentries%, percentage of open-arm entries; Otime%, percentage of open-arm time; TST, tail suspension test; HPA, hypothalamic-pituitary-adrenal; CRH, corticotropin-releasing hormone; TLRs, toll-like receptors
